# *Oxalis simplicifolia* (Oxalidaceae), an unusual new unifoliolate species from the Marquesas Islands (French Polynesia)

**DOI:** 10.3897/phytokeys.4.1604

**Published:** 2011-07-12

**Authors:** David H. Lorence, Warren L. Wagner

**Affiliations:** 1 National Tropical Botanical Garden, 3530 Papalina Road, Kalaheo, HI 96741 USA; 2 Department of Botany, MRC-166, National Museum of Natural History, Smithsonian Institution, P.O. Box 37012, Washington, DC 20013-7012

**Keywords:** Conservation, French Polynesia, Marquesas Islands, *Oxalis*, Oxalidaceae, unifoliolate

## Abstract

*Oxalis simplicifolia* Lorence & W. L. Wagner **sp. nov.**, a new species from the Marquesas Islands (Ua Huka) is described and illustrated. It differs from the other Marquesas species, *Oxalis gagneorum*, in having simple, unifoliolate glabrous leaves, minutely glandular-puberulent calyx lobes, shorter corolla lobes, erect capsules, and smaller seeds. As its habitat is under serious threat from human impact, feral animals, and weeds, we conclude this new species should be added to the IUCN Red List as critically endangered (CR).

## Introduction

Intensive botanical exploration of the Marquesas Islands (French Polynesia) for the Vascular Flora of the Marquesas Islandsand Flore de la Polynésie française projects has resulted in numerous additional new collections from these islands. During the collecting expeditions for the current Vascular Flora of the Marquesas Islands project under the direction of David H. Lorence and Warren L. Wagner (Wagner and Lorence 1997) a unique unifoliolate species of *Oxalis* was collected on the island of Ua Huka by Steve Perlman and Ken Wood of the National Tropical Botanical Garden. An analysis of the conservation status of this new species reveals it should be included as a critically endangered (CR) species in the IUCN Red List.

*Oxalis* is a cosmopolitan genus of approximately 500 to 700 species with its greatest diversity in South America and the African Cape region (Mabberley 2008). Previously only a single native species of *Oxalis* was known from the Marquesas: *Oxalis gagneorum* Fosberg & Sachet, a small shrub 30–40 cm tall endemic to the islands of Eiao, Hiva Oa, Tahuata, and Fatu Hiva. The naturalized herbaceous species *Oxalis corniculata* L. also occurs in suitable habitat on most of the main islands.

## Methodology

All measurements given herein are taken from dried herbarium specimens, although certain features such as shapes were supplemented with information from alcohol-preserved flowers and fruits, field notes, and digital photos. Measurements are presented in the descriptions as follows: length × width, followed by units of measurement (mm or cm). All specimens cited in this paper have been seen by the authors. The area of occupancy (distribution) for this species was calculated using herbarium collection data and field observations, and the conservation status is proposed following the IUCN Red List Category criteria (IUCN 2001; see also www.iucnredlist.org/info/categories_criteria2001).

## Systematics

### Oxalis simplicifolia

Lorence & W. L. Wagner sp. nov.

urn:lsid:ipni.org:names:77112690-1

http://species-id.net/wiki/Oxalis_simplicifolia

Figs 1 2 

#### Latin.

Ad Oxalidem gagneorum Fosberg & Sachet affinis sed in foliis simplicibus unifoliolatis glabris, in laminis ovatis vel late ovatis subpalmate nervatis, in lobis calycis minute glandulo-puberulis, in lobis corollae brevioribus 8-12 mm longis, in staminibus 5-8 mm longis, in capsulis maturis rectis et in seminis minoribus 0.8-0.9 × 0.5 mm differt.

#### Type.

**MARQUESAS ISLANDS:** Ua Huka: Hanahouua valley, back of valley below cliff walls, 457 m elevation, 8°54.47S, 139°30.89W, 26 June 2004, S. Perlman & K. R. Wood 19072 (holotype: PTBG-041184!; Isotypes: P!, PAP!, US!).

#### Description.

*Perennial woody herbs or subshrubs* 20–50 cm tall, stems prostrate or sprawling to erect, branching from near base, with sparse lateral branches, glabrous or new growth sparsely pilose, mature twigs 2–3 mm diam, bark smooth, reddish brown to dark brown, with tufts of pilose hairs at thickened, persistent leaf bases. *Leaves* simple, spirally arranged; blade dark green above, yellow-green below, firm and moderately coriaceous when fresh, chartaceous when dry, glabrous, (15–) 20–47 × (12–)18–37 mm ovate to broadly ovate, base obtuse to rounded or truncate, apex obtuse, tip usually emarginate, venation subpalmate with 1–2(–3) pairs of secondary veins from base and 2–3 pairs along midrib above, venation raised and visible to 3° above and to 4° beneath; margin thin, plane; petiole (20–) 25–45 × 0.6–0.8 mm, sparsely scattered pilose or glabrescent, flattened, adaxially sulcate, distally with slight pulvinus. *Inflorescences* axillary near ends of branches, cymose, 5–13–flowered, 5–8 cm long, peduncles 4–5 cm long, terminating in apical flower and two monochasial lateral branches 18–40 mm long each with 2–6 flowers, bracts linear-subulate, 1–2 × 0.3–0.4 mm, sparsely puberulent with acicular and scattered glandular-tipped trichomes. *Flowers* (long-styled morph seen) with 5 calyx lobes 5–7 × 1.7–2 mm, narrowly ovate-oblong, 6–8-veined, apex acute, both surfaces minutely glandular-puberulent with capitate trichomes; petals yellow, 10–12 × 5 mm, narrowly obovate to oblong-elliptic, 7–9-veined, apex obtuse to rounded; stamens 10, in two series, the longer 7–8 mm long, the shorter 5.5–6 mm long, filaments connate basally, anthers broadly ellipsoid, 0.4–0.5 mm long, reniform to subcircular; gynoecium 10-14 mm long, with ovary 6–7 mm long, narrowly ovoid-cylindrical, externally glabrous, beak 4–5 mm long, styles 5, 1–2.5 mm long, stigmas slightly thickened, papillose, not bifid. *Fruits* ovoid-cylindrical, 10–11 × 2–2.5 mm, at maturity apparently straight and not twisting, externally glabrous, carpels 5, villous within, seeds ca. 40. *Seeds* ellipsoid, compressed, 0.8–0.9 × 0.5 mm, surface shiny, brown, rugose.

**Figure 1.**
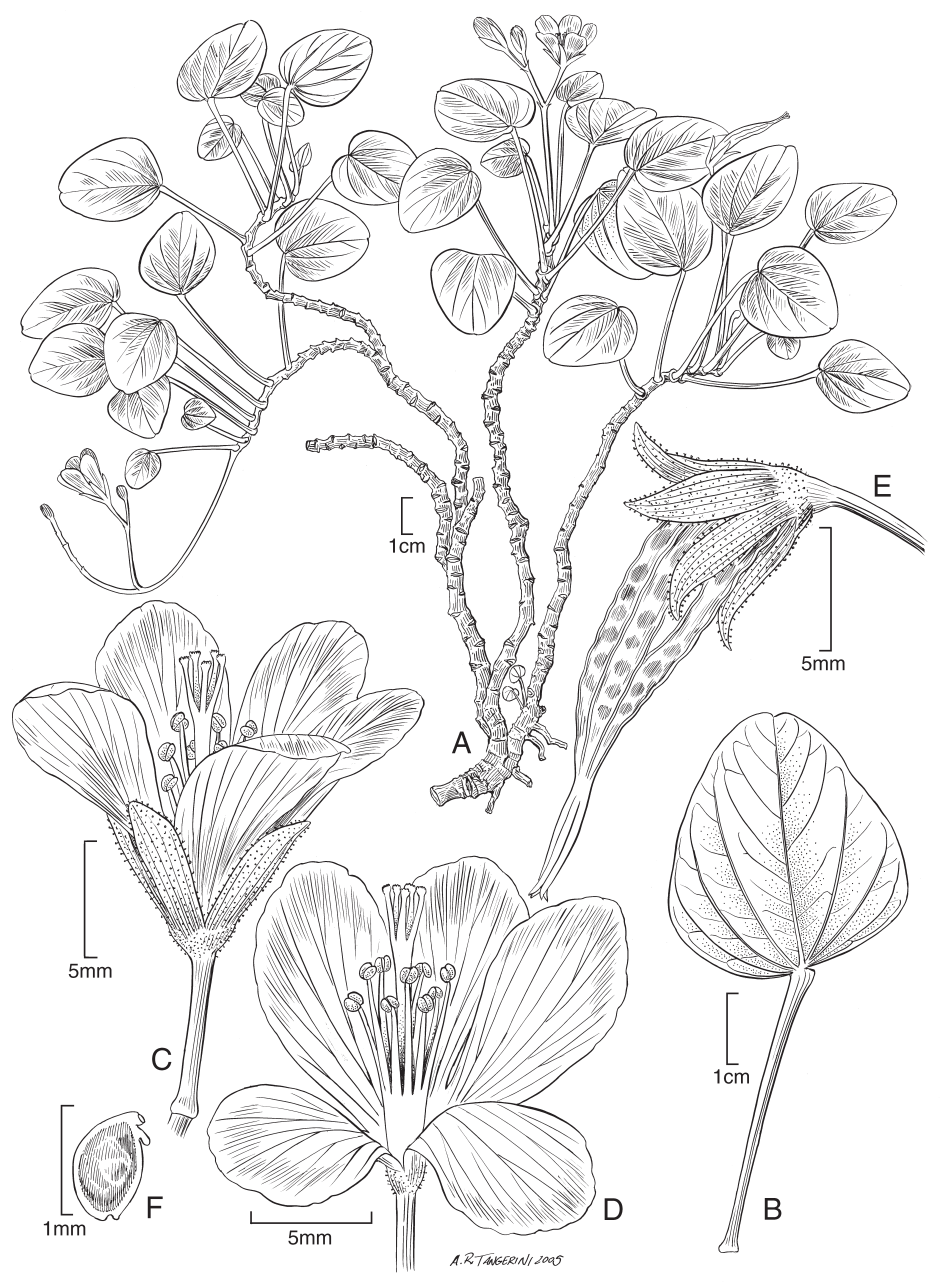
*Oxalis simplicifolia* Lorence & WL Wagner **A** habit **B** leaf **C, D** flowers **E** fruit **F** seed. Drawn from the type collection (Perlman & Wood 10972) and field images.

#### Distribution.

Known only from Ua Huka, Marquesas Islands.

#### Ecology.

**Figure 2.**
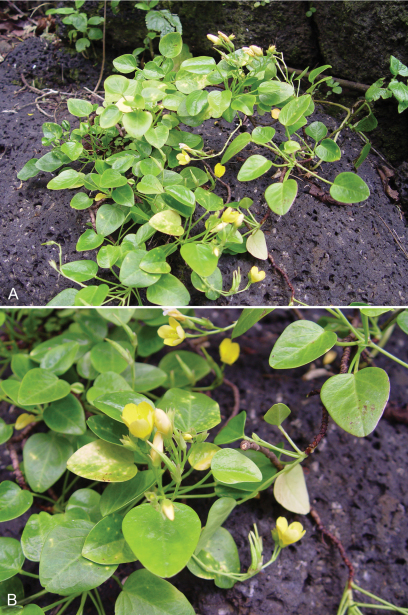
Field images of *Oxalis simplicifolia* **A** habit **B** stems with flowers and fruits (Perlman & Wood 10972).

Known only from two localities on Ua Huka, this new species occurs in shrubby and herbaceous vegetation on vertical basalts cliff above a mesic to wet lowland forest zone with *Freycinetia impavida* (Gaudich. ex Hombr.) B.C. Stone, *Hibiscus tiliaceus* L.*, Pandanus tectorius* Parkinsonр and *Pisonia grandis* R. Br. Plants grow scattered on cliffs rooting in rock crevices (Figures 2A, B). *Oxalis gagneorum* occurs sympatrically or nearly so with *Oxalis simplicifolia* at both Hanahouua (Perlman and Meyer 19748) and Hane/Hokatu (Wood & Meyer 10530, 10551).

#### Etymology.

The specific epithet refers to the simple, unifoliolate leaves.

#### Conservation status.

Following the criteria and categories of IUCN (2001) *Oxalis simplicifolia* is assigned a preliminary status of Critically Endangered (**CR**): B2a, B2b (i–iii); D): B2: total area of occupancy less than 10 km2 (ca. 5 km2). B2a, two populations known; b (i–iii), habitat continuing decline inferred. D, population estimated to number fewer than 250 individuals. The suitable habitat for *Oxalis simplicifolia* on Ua Huka (ca. 83 km2) is indicated as an endangered environment, threatened by feral animals and invasive plants, reducing the extent of the forest. Estimated population size is ca 100+ plants at the Hanahouua locality (Perlman & Meyer 19748), and “scattered” individuals were noted by the collectors at the Hane/Hokatu locality (Wood & Meyer 10530, 10551).

#### Specimens examined.

**Marquesas Islands:** Ua Huka: Hanahouua valley, back of valley on ridge between Hanahouua and Hanalei, 8°54.47S, 139°30.87W, 488 m, 28 July 2005, S. P. Perlman, J.-Y. Meyer 19748 (PTBG); Hane/Hokatu cliff, zone, 520 m, 11 Dec 2003, K. R. Wood, J.-Y. Meyer 10512 (PAP, PTBG, US).

#### Discussion.

Although the majority of *Oxalis* species have palmately compound leaves with three (rarely to nine) leaflets, several taxa with unifoliolate leaves occur in South America in subgenus *Thamnoxys* (Lourteig 1994). At least three South African taxa are also unifoliolate: *Oxalis monophylla* L., *Oxalis salteri* L.Bolus, and *Oxalis flava* L. var. *unifoliolata* Dreyer & Oberl. (Dreyer et al. 2010). These are small, bulbous, acaulescent plants with white or yellow flowers apparently unrelated to *Oxalis simplicifolia*. Certain species, e.g. *Oxalis renifolia* Kunth and a few other South American taxa can have one and three leaflets on the same branch (E. Emshwiller, pers. comm. 2009). Following Lourteig’s (1994, 2000) monograph *Oxalis simplicifolia* keys to subgenus *Monoxalis* (leaves simple, 1-foliolate, stigmas linguiform) which consists of two herbaceous species from the southwestern United States and Mexico (*Oxalis dichondrifolia* A. Gray and *Oxalis robusta* Kunth), neither of which bears any morphological similarity to *Oxalis simplicifolia*.

This new species may be related to the Marquesas endemic *Oxalis gagneorum*, from which it differs by its glabrous simple, unifoliolate leaves with ovate to broadly ovate blades having subpalmate venation of one to three basal vein pairs, minutely glandular puberulent calyx lobes, shorter corolla lobes 8–12 mm long, shorter stamens 5–8 mm long, capsules not twisting at maturity (they often twist in *Oxalis gagneorum*), and smaller seeds 0.8–0.9 × 0.5 mm (Table 1). Fosberg and Sachet (1981: 3–5, Fig. 1) stated that the relationships of *Oxalis gagneorum* were obscure and suggested that it may be related to *Oxalis novaecalidoniae* Kunth & Schlechter, a species belonging to section *Caledonicae* (= section *Neocalidonicae*), but that mature seeds were needed for more accurate placement. Lourteig (2000) placed *Oxalis gagneorum* in section *Rhombifoliae* along with several neotropical species characterized by “lianoid” transversely striate stems [a character not apparent in material of either Marquesan species studied by us] and trifoliolate leaves with oblong to rhomboidal leaflets and lacking stipules, but expressed doubt as to its relationships due to the poor material available for study (i.e., lacking stigmas and seeds). Unfortunately, it has not been possible to obtain DNA sequences from samples of either Marquesan species thus far (E. Emshwiller, pers. comm. 2009), and consequently their phylogenetic relationships remain unclear.

**Table 1.** Distinguishing morphological features of *Oxalis gagneorum* and *Oxalis simplicifolia*.

Character	*Oxalis gagneorum*	*Oxalis simplicifolia*
Height (m)	0.3–1.2	0.2–0.5
Stem pubescence	young growth pilose	glabrous or sparsely pilose
Leaflet pubescence	pilose-strigose below	glabrous
Leaflet number	3	1
Leaflet shape	broadly obovate or oblong-elliptic	ovate to broadly ovate
Leaflet blade length (mm)	35	(20–) 25–47
Leaf blade width (mm)	26	(12–) 18–37
Venation	pinnate	subpalmate
Secondary vein pairs	5–7	1–2 (–3) basal, 2–3 above
Flowers per inflorescence	3–5	5–13
Calyx lobe shape	ovate	ovate-oblong
Calyx lobe length (mm)	4–6	5–7
Calyx lobe width (mm)	2–3	1.7–2
Calyx pubescence	sparsely pilose	minutely glandular puberulent
Corolla lobe length (mm)	12–35	8–12
Corolla lobe width (mm)	3–5	5
Corolla lobe shape	narrowly obovate to spathulate, clawed	narrowly obovate to oblong-elliptic
Shorter stamen length (mm)	11–14	5–5.6
Longer stamen length (mm)	14–16	7–8
Fruit shape	Broadly cylindrical, twisting at maturity	Ovoid-cylindrical, not twisting at maturity
Fruit length (mm)	9–15	10–11
Fruit width (mm)	3–4	2–2.5
Seed length (mm)	1.3–1.4	0.8–0.9
Seed width (mm)	0.8–1.2	0.5
Seed surface	shiny, brown, rugose	shiny, brown, rugose

*Oxalis* species often have different floral morphs, frequently tristylous or sometimes distylous (Weller et al. 2007). Examination of material of *Oxalis gagneorum* revealed most flowers appear to be homostylous with styles about equaling the stamens. Due to a paucity of flowering collections of *Oxalis simplicifolia*, only the long-styled floral morph with both whorls of stamens shorter than the style is known (illustrated in Fig. 1). There may be several possible explanations for this: either *Oxalis simplicifolia* might be a clonally reproducing species with a single morph (as are several *Oxalis* species in Mexico), or it could be an autogamous species that reproduces sexually but has only a single morph (S. Weller, pers. comm. 2009). Further collections and field studied are clearly necessary to resolve this question.

## Supplementary Material

XML Treatment for Oxalis simplicifolia
